# Transcriptomic and metabolomic analyses reveal *OsACL-A2* coordinates light signaling, stress responses, and metabolic changes in rice

**DOI:** 10.3389/fpls.2026.1789851

**Published:** 2026-04-10

**Authors:** Ziqiang Chen, Lan Kong, Huaqing Liu, Gang Li, Dagang Tian, Yue Wang, Yarong Lin, Jingwan Yan, Xinrui Guo, Changquan Hu, Shaohua Yang

**Affiliations:** 1 Biotechnology Research Institute, Fujian Academy of Agricultural Sciences, Fuzhou, China; 2 Crop Research Institute, Fujian Academy of Agricultural Sciences, Fuzhou, China

**Keywords:** cell death, lesion mimic mutants, metabolome, transcriptome, phenylpropanoid metabolism, rice

## Abstract

**Introduction:**

Lesion mimic mutants (LMMs) spontaneously develop defense-related cell death lesions, serving as ideal models for studying plant immunity. However, the metabolic mechanisms underlying light-dependent lesion formation remain poorly understood.

**Methods:**

To address this, we performed comparative transcriptomic and metabolomic analyses on wild-type rice and the lmm64 mutant, which carries a novel allele of *OsACL-A2*, under light and dark conditions.

**Results:**

The *lmm64* mutant develops light-dependent lesions accompanied by ROS accumulation. Transcriptomic analysis revealed 750 lesion-associated genes specifically altered under light, which were enriched in stress responses, light signaling, carbon metabolism, and secondary metabolism. Stress-responsive genes were predominantly upregulated. Multiple light signaling components (e.g., *HY5*, *PIF3*) showed marked transcriptional changes. Meanwhile, acetyl-CoA metabolism-related genes (*ACS*, *PDHA1*, *ACO*) were upregulated, likely as a compensatory response to *OsACL-A2* dysfunction, while glycolytic genes (*PFK1*, *PK*, *ADH*) were downregulated. At the metabolic level, phenolic compounds including flavonoids and phenolic acids were significantly reduced, consistent with the downregulation of key phenylpropanoid/flavonoid biosynthetic genes (e.g., *CHS*, *CHI*, *F3'H*, *4CL2*, *HCT*).

**Conclusion:**

These findings reveal that *OsACL-A2* integrates light signaling, stress responses, and metabolic reprogramming to orchestrate light-dependent lesion formation in rice, expanding its known functions.

## Introduction

LMMs spontaneously develop necrotic lesions resembling hypersensitive response (HR) symptoms in the absence of pathogen infection. These mutants have been identified across diverse plant species, including Arabidopsis, rice, barley, wheat, and maize (Chen et al., 2025; Li et al., 2020, 2022). Lesion formation in LMMs is typically accompanied by reactive oxygen species (ROS) accumulation, programmed cell death (PCD), and chloroplast dysfunction (Zhang et al., 2022; Kang et al., 2021; Lorrain et al., 2003). The underlying mechanisms are complex, involving both internal genetic factors and external environmental cues. Therefore, LMMs provide valuable models for dissecting the molecular events leading to HR-like cell death and understanding the interplay between defense activation and cellular homeostasis.

To date, numerous lesion mimic mutants and corresponding genes have been identified and cloned across various plant species, particularly in rice, with over 60 such genes reported (Rao et al., 2021; Chen et al., 2025). These genes are involved in diverse biological processes, such as tryptamine metabolism (*SL*), tetrapyrrole metabolism (*CPOX*, *LEN1*), protein ubiquitination (*spl11*), Protein processing in endoplasmic reticulum (*spl7*) and MAPK signaling (*spl3*) (Fujiwara et al., 2010; Sun et al., 2011; Ma et al., 2020; Ishikawa, 2005; Zeng et al., 2004; Yamanouchi et al., 2002; Wang et al., 2015). Despite the diversity of causal mutations, the associated immune processes in lesion mimic mutants have been extensively characterized, typically involving disruption of ROS homeostasis, oxidative stress, and PCD (Chen et al., 2025; Tian et al., 2025). Simultaneously, many lesion mimic mutants exhibit activated defense responses, marked by upregulated expression of pathogenesis-related (PR) genes and increased levels of defense hormones such as salicylic acid (SA) and jasmonic acid (JA), conferring enhanced resistance to pathogens including *Xanthomonas oryzae pv. oryzae* and *Magnaporthe oryzae* (Fang et al., 2025; Tian et al., 2020; Wang et al., 2025).

Transcriptomic and metabolomic analyses of lesion mimic mutants have revealed extensive reprogramming of metabolic and defense pathways. For instance, the rice *spl39* mutant exhibits upregulation of hormone signaling and defense pathways with elevated SA, JA, auxin, and cytokinin levels due to diterpenoid biosynthetic gene cluster deletion (Fang et al., 2025). Similarly, some mutants show a shift from growth-oriented to defense-oriented metabolism, characterized by upregulation of phenylpropanoid and terpenoid pathways, suppression of photosynthesis-related genes, and alterations in phytohormone, fatty acid, and sugar metabolism (Mu et al., 2021; Li et al., 2024). These coordinated changes may be associated with spontaneous cell death and enhanced disease resistance.

The lesion phenotype in LMMs is also modulated by environmental factors, particularly light and temperature. Light intensity and photoperiod influence lesion development in multiple mutants, as demonstrated by light-shielding experiments that suppress lesion development in several LMMs (Ueno et al., 2003; Ishikawa, 2005). Temperature sensitivity has also been documented, with *spl7* requiring heat stress (>35 °C) and *ubc13* exhibiting low-temperature-induced lesion formation (Cui et al., 2021; Wang et al., 2019; Yamanouchi et al., 2002). These environmental regulations highlight the complex interplay between genetic mutations and external cues in lesion development.

ATP-citrate lyase (ACL) catalyzes the cleavage of citrate to generate acetyl-CoA and oxaloacetate, thereby providing the cytosolic acetyl-CoA pool essential for fatty acid synthesis, histone acetylation, and secondary metabolism (Xu et al., 2023). The role of ACL in lesion mimic mutants has been established through independent rice studies. Ruan et al. (2019) first identified the *OsACL-A2* lesion mimic mutant (*spl30-1*), showing reduced ACL activity, elevated ROS, and upregulated defense genes via SPL30 protein degradation through the ubiquitin-26S proteasome system. Subsequently, Duan et al. (2025) characterized another *OsACL-A2* mutant, revealing a distinct mechanism involving iron homeostasis disruption, reduced leaf iron, increased citric acid accumulation, and iron deficiency-induced chlorosis/necrosis rescued by iron supplementation. Despite these advances, how ACL dysfunction triggers cell death and integrates environmental signals, particularly light, remains unclear. In this study, we sought to determine the molecular bases of *OsACL-A2* mutation-induced lesion formation under light conditions through integrated transcriptomic and metabolomic analyses of *lmm64* mutant, thereby providing new insights into the gene expression and metabolite changes associated with lesion formation and defense-related traits in rice.

## Materials and methods

### Plant material and growth conditions

The *lmm64* mutant was isolated from an EMS-mutagenized population of the indica maintainer line ‘MingHui 86’ (Wild type, WT), and the lesion mimic phenotype was stably inherited over generations. For gene mapping, *lmm64* mutant was crossed with the polymorphic restorer line ‘9311’ to generate F_1_ hybrids. An F_2_ mapping population was then obtained by self-pollinating the F_1_ plants.

All plants were cultivated in the experimental field of the Biotechnology Institute, Fujian Academy of Agricultural Sciences, Fuzhou, China, under standard local agronomic practices during the natural rice growing season (May to October). Plants were exposed to natural sunlight and natural photoperiod (approximately 13–14 h light/10–11 h dark depending on the growth stage). The temperature approximately ranged from 25-35 °C during the day and 20–30 °C at night. For light-shielding experiments, specific leaf sections were covered with aluminum foil for 3 days prior to lesion appearance while maintaining otherwise identical field conditions.

### Phenotypic characterization and agronomic trait evaluation

Leaf phenotypes were documented at different developmental stages. At maturity, ten plants each of the wild type and *lmm64* mutant were randomly sampled to evaluate key agronomic traits, including effective panicle number, grain number per panicle, seed setting rate, and 1000-grain weight.

### Physiological and biochemical analyses

Cell death and hydrogen peroxide (H_2_O_2_) accumulation in leaves of WT and *lmm64* mutant plants were assessed histochemically using trypan blue and 3,3′-diaminobenzidine (DAB) staining, respectively, according to the methods described by Mu et al. (2021) with minor modifications. Briefly, for cell death visualization, samples were stained with trypan blue solution (2.5 mg/mL in lactic acid:phenol:glycerol:water = 25:23:25:27, v/v), boiled for 2 min, and incubated overnight, followed by chlorophyll clearance in chloral hydrate (2.5 g/mL) for 3 days. For H_2_O_2_ detection, samples were incubated in 1 mg/mL^-1^ DAB (pH 3.8) in darkness for 8 h before chlorophyll was removed with 90% ethanol. To assess the light dependence of lesion formation, leaf sections of both genotypes were shaded with aluminum foil for 3 days prior to lesion appearance.

### Genetic mapping

For genetic mapping, genomic DNA was extracted from F_2_ plant leaves using the hexadecyltrimethylammonium bromide (CTAB) method (Richards et al., 2001). Insertion/deletion (InDel) markers, developed in our laboratory by comparing the genomic sequences of ‘MingHui86’ and ‘9311’, were utilized for subsequent genotyping and linkage analysis.

To assess the conservation of the Gly306 residue, we utilized the RiceVarMap database (http://ricevarmap.ncpgr.cn/), which compiles genomic variation data from 4,726 diverse rice cultivars worldwide. The genomic sequence of *OsACL-A2* (*LOC_Os12g37870*) was queried using the “Search for Variations in Gene” function. All variants within the gene region, including the exon encoding Gly306, were examined.

### Preparation of plant material for transcriptomic and metabolomic analyses

Samples for integrated transcriptomic and metabolomic analyses were collected from shaded sections and light-exposed lesioned sections of both WT and *lmm64* mutant plants. The experimental design included four sample groups. Shaded sections of WT were designated as WT-D, shaded sections of *lmm64* as *lmm64-D*, light-exposed lesioned sections of WT as WT-L, and light-exposed lesioned sections of *lmm64* as *lmm64-L*. Each group contained three biological replicates, with each replicate consisting of leaves pooled from three independent plants. Following collection, all samples were immediately homogenized in liquid nitrogen and divided into two equal aliquots for parallel transcriptomic and metabolomic analyses.

### RNA sequencing and bioinformatic analysis

Total RNA from 12 samples was extracted and used for sequencing library construction following the manufacturer’s protocol. The resulting libraries were quality-controlled and sequenced on an Illumina HiSeq™ 2000 platform, generating 150-bp paired-end reads. After removal of low-quality sequences and adapter contamination, the clean reads were aligned to the rice reference genome (MSU Rice Genome Annotation Project Release 7) for subsequent analysis.

Transcript accumulation levels were quantified using the FPKM (fragments per kilobase of transcript per million fragments mapped) method. Differentially expressed genes (DEGs) were identified using DESeq2 with a false discovery rate (FDR) < 0.01 and absolute fold-change ≥ 2 (Love et al., 2014). Gene Ontology (GO) enrichment and Kyoto Encyclopedia of Genes and Genomes (KEGG) pathway analyses were performed using the Metware Cloud platform (https://cloud.metware.cn), a free online bioinformatics tool.

### Metabolomic analysis

A total of 12 samples, with three biological replicates per group, were collected for comprehensive metabolite profiling. Sample preparation and UPLC-MS/MS analysis were performed by Metware Biotechnology Co., Ltd (Wuhan, China). Metabolite profiling was conducted on a UPLC-ESI-MS/MS system consisting of an ExionLC™ AD UPLC unit (SCIEX) coupled with a Q TRAP^®^ 4500 mass spectrometer (Applied Biosystems) equipped with an ESI Turbo Ion-Spray interface. The mass spectrometer was operated in both positive and negative ionization modes. System control and data acquisition were performed using Analyst^®^ 1.6.3 software (SCIEX). Raw MS data were processed for peak detection, alignment, and integration using the Metware Cloud platform. Metabolites were identified by matching MS/MS spectra against the Metware Database (MWDB) and public spectral libraries.

Differentially accumulated metabolites (DAMs) between two-group comparisons were identified based on the following criteria: variable importance in projection (VIP) > 1, |log_2_FC| ≥ 1, and p < 0.05 (determined by hypergeometric test). For KEGG enrichment analysis, DAMs were annotated using the KEGG Compound database (http://www.kegg.jp/kegg/compound/) and mapped to the KEGG Pathway database (http://www.kegg.jp/kegg/pathway.html). Pathway enrichment significance was assessed using metabolite sets enrichment analysis (MSEA) with hypergeometric test p-values. All statistical analyses and visualizations, including Venn diagrams, heatmaps, and enrichment analysis circle plots, were generated using the Metware Cloud platform.

### Gene expression analysis

To examine gene expression differences between the wild type and *lmm64* mutant under shaded and normal light conditions, leaf samples exhibiting distinct phenotypes were collected. Total RNA was isolated using TRIzol reagent (Invitrogen) following the manufacturer’s instructions. Subsequently, 1 μg of RNA was reverse transcribed into cDNA using HiScript III 1st Strand cDNA Synthesis Kit (+gDNA wiper) (Vazyme). Quantitative RT-PCR amplifications were carried out on an ABI Sequence Detection System (Applied Biosystems) with Taq Pro Universal SYBR qPCR Master Mix (Vazyme). The relative expression levels of target genes were calculated using the 2-ΔΔCT method, with OsACTIN serving as an internal reference (Ruan et al., 2019). All primer sequences used in this study are listed in **Supplementary Table 1**. The experiment included three biological replicates, with each replicate consisting of leaves mixed from three independent plants.

## Results

### The light-dependent mutant *lmm64* is caused by a mutation in *OsACL-A2*

**Figure 1:**
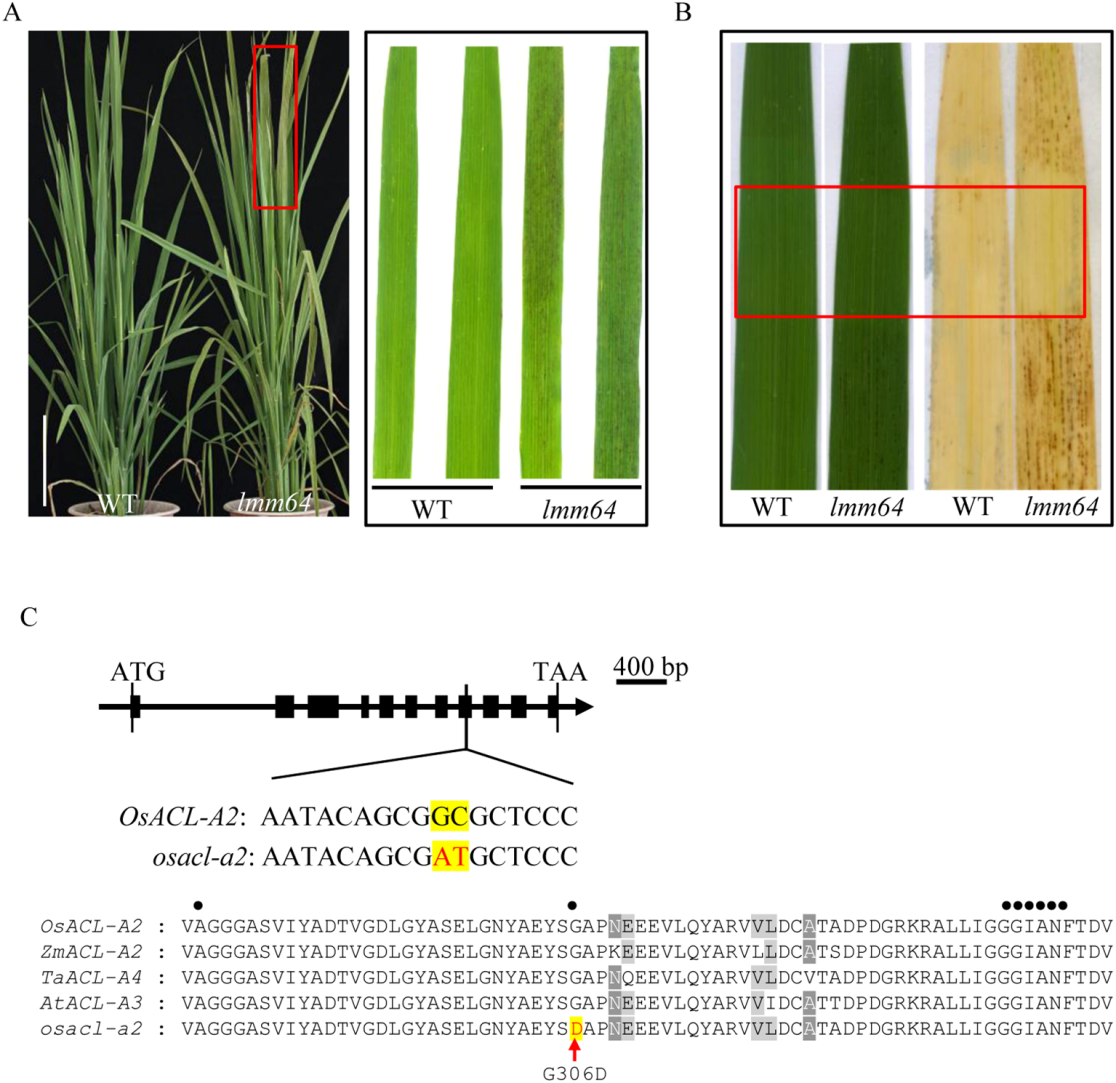
Phenotypic characterization and gene identification of *lmm64* mutant. **(A)** Plant phenotypes of WT and *lmm64* at the late tillering stage. Scale bar =20 cm. **(B)** Light-shielding treatment followed by DAB staining in WT and lmm64. **(C)** Gene structure of the candidate gene *LOC_Os12g37870*. Black boxes represent exons. A point mutation (GC to AT) was identified in the eighth exon. Alignment of amino acid sequences between WT andlmm64 showing a Gly306Asp substitution within the conserved citrate-binding domain, indicated by a solid circle.

The *lmm64* mutant, displaying reddish-brown spots on leaves from the late tillering stage under field conditions, was obtained from an ethyl methanesulfonate (EMS) mutant library (**Figure 1A**). Histochemical analysis showed that, compared with WT plants, *lmm64* lesions exhibited strong Trypan blue and positive DAB staining, indicating cell death and ROS accumulation, respectively (**Supplementary Figures 1A, B**). Critically, lesion formation and ROS accumulation were light-dependent, as demonstrated by light-shielding experiments (**Figure 1B**). The mutant also displayed a significant reduction in 1000-grain weight, without apparent effects on other major agronomic traits (**Supplementary Figure 1C**).

Map-based cloning and sequencing analysis identified a single GC-to-AT transition in the eighth exon of *OsACL-A2* (*LOC_Os12g37870*), resulting in a Gly306Asp substitution within the conserved citrate-binding domain (**Figure 1C**; **Supplementary Figure 1D**). The gene identified in this study is the same as previously reported by Ruan et al. (2019) and Duan et al. (2025). No sequence variations were detected in the other five genes within the mapping interval. Analysis of the RiceVarMap database further revealed that the Gly306 residue is highly conserved across diverse rice cultivars (**Supplementary Table 2**), supporting its functional importance.

### Transcriptome analysis reveals constitutive defense activation and metabolic alterations under light in *lmm64* mutant

To gain insights into the systemic alterations underlying the light-responsive cell death phenotype in *lmm64* mutant, we firstly performed comparative transcriptomic analysis (RNA-Seq) on both *lmm64* and WT plants under light (designated as *lmm64-L* and WT-L) and dark (*lmm64-D* and WT-D) growth conditions. Our sequencing generated 148.09 Gb of high-quality data, with all samples yielding >6 Gb of clean reads and excellent quality metrics (Q30 scores: 92.10-95.33%) (**Supplementary Table 3**). Biological replicates showed strong correlation (**Supplementary Figure 2A**), indicating the high reliability of the dataset. We identified differentially expressed genes (DEGs) in four comparisons under stringent criteria (|log_2_FC| ≥ 1, FDR ≤ 0.05): *lmm64-L* vs WT-L showed 1969 upregulated and 1585 downregulated genes; *lmm64-D* vs WT-D had 3088 upregulated and 2317 downregulated; WT-D vs WT-L exhibited 3064 upregulated and 2066 downregulated; and *lmm64-D* vs *lmm64-L* displayed 3909 upregulated and 3301 downregulated (**Supplementary Figure 2B**). Notably, the *lmm64-L* vs WT-L comparison yielded fewer DEGs than other comparisons, likely because the lesion phenotype results from relatively specific transcriptional changes in key pathways rather than widespread transcriptional perturbations.

**Figure 2:**
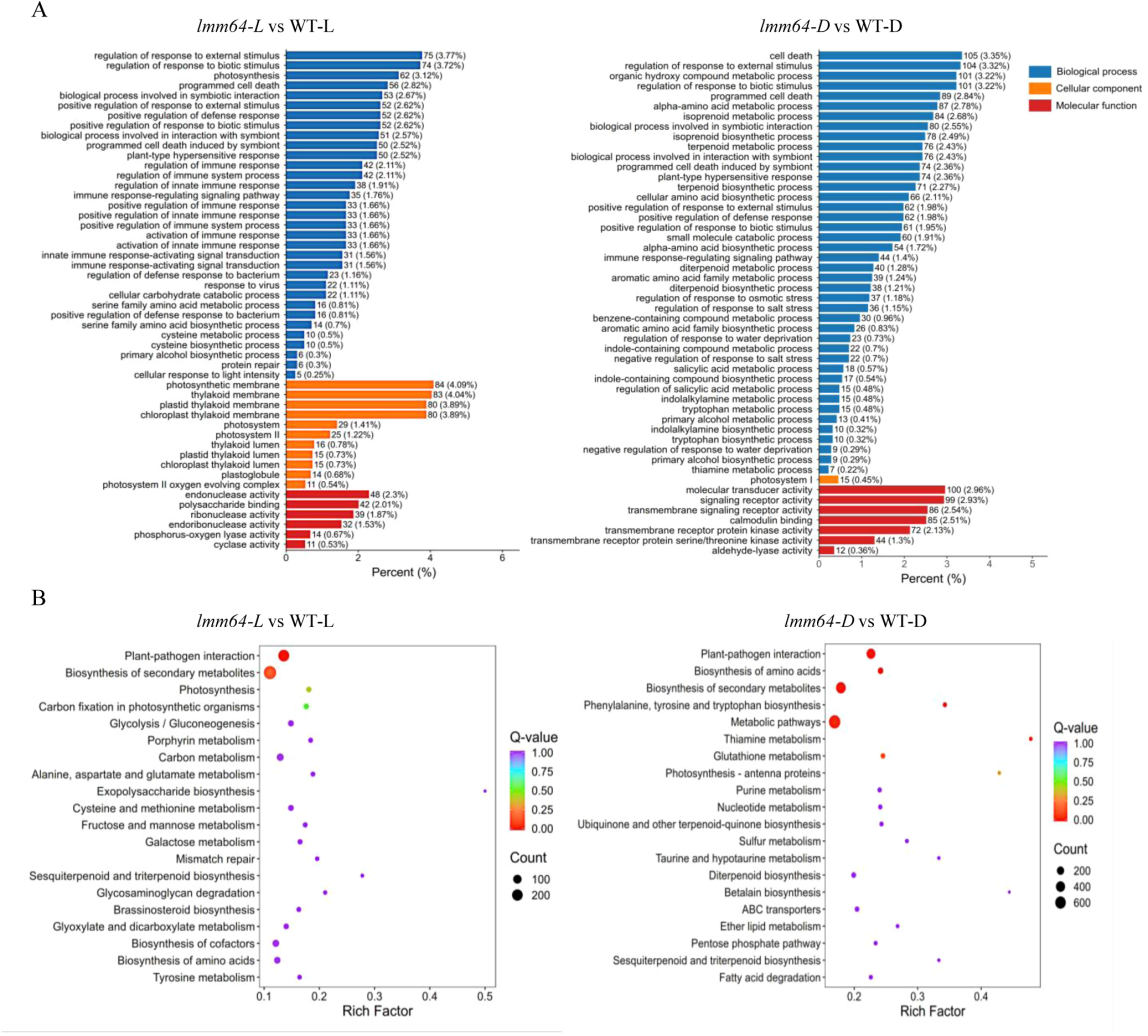
Transcriptome analysis reveals constitutive defense activation and metabolic alterations under light in *lmm64*. **(A)** Top 50 significantly enriched GO terms from comparisons of *lmm64-L* vs WT-L and *lmm64-L* vs *lmm64-D*. Values indicate the number of DEGs annotated to the term, with the ratio to total annotated DEGs in parentheses. **(B)** Top 20 significantly enriched KEGG pathways from the same comparisons. The rich factor represents the degree of enrichment; point size corresponds to the number of DEGs, and color intensity reflects the enrichment significance (-log10(P-value)).

To explore the functional implications of DEGs, we performed GO and KEGG enrichment analyses on comparisons between WT and *lmm64* under light and dark conditions. GO analysis revealed that DEGs under both conditions were commonly enriched in defense and cell death-related processes (e.g., ‘defense response’, ‘programmed cell death’), while photosynthesis-related terms, including ‘photosynthesis’, ‘photosynthetic membrane’, and ‘thylakoid membrane’, were enriched exclusively under light (**Figure 2A**). KEGG analysis showed that pathways such as ‘plant-pathogen interaction’ and ‘biosynthesis of secondary metabolites’ were enriched under both conditions, whereas energy metabolism pathways, including ‘photosynthesis’, ‘carbon fixation’, and ‘carbon metabolism’, were more enriched under light (**Figure 2B**). These findings indicate that *lmm64* mutation causes constitutive activation of defense and cell death programs regardless of light, while energy metabolism pathways are specifically altered under light conditions, coinciding with lesion formation.

### Identification of 750 genes specifically associated with light-dependent lesion formation

**Figure 3:**
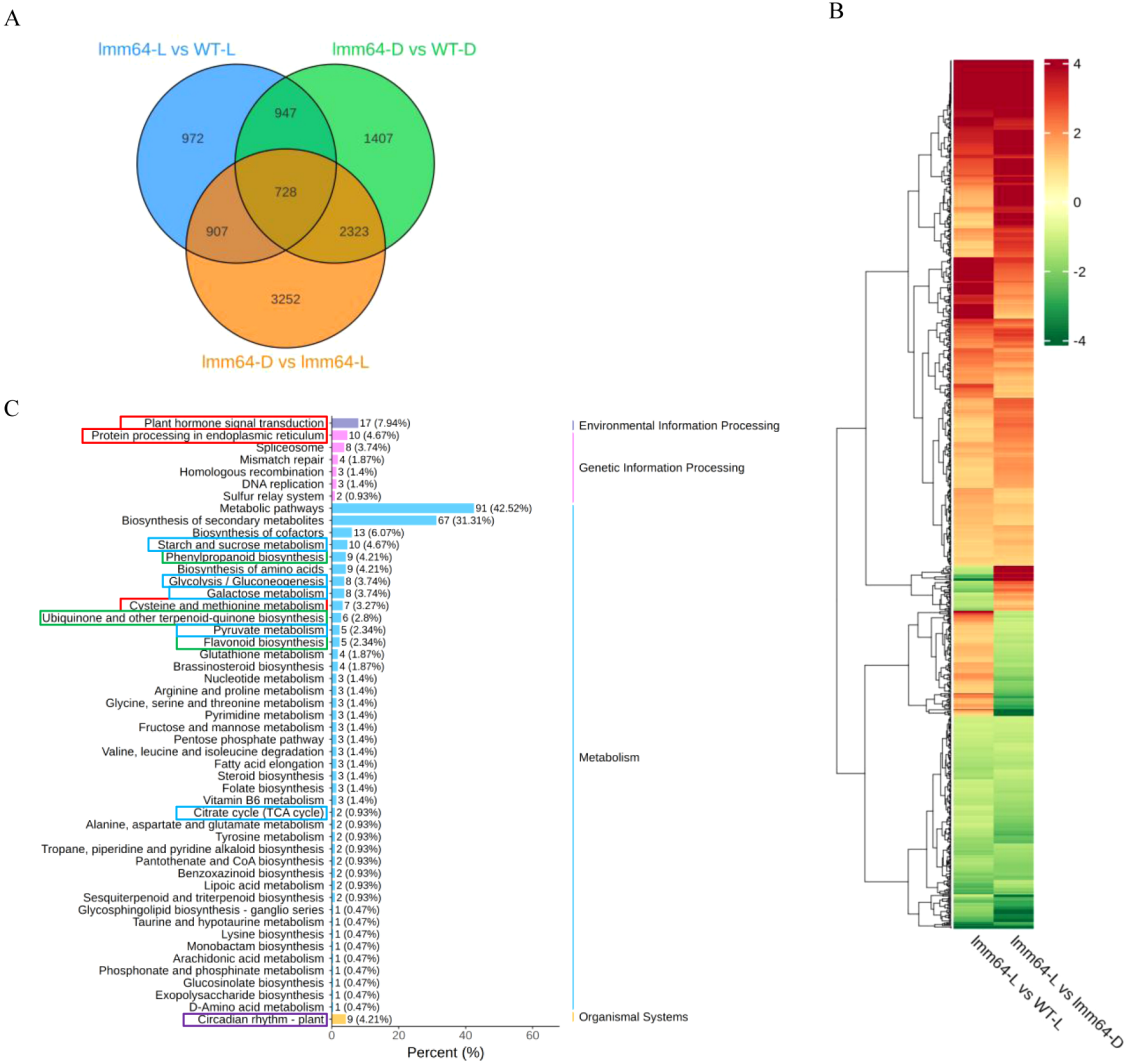
Identification of DEGs associated with lesion formation under light in *lmm64*. **(A)** Venn diagram of DEGs identified from comparisons involving lesioned (*lmm64-L*) and non-lesioned samples. **(B)** Hierarchical clustering of DEGs in lesioned versus non-lesioned samples. Color intensity reflects the magnitude of expression change (red: up-regulated; green: down-regulated). **(C)** KEGG enrichment analysis of lesion-associated DEGs. Values indicate the number of DEGs in each pathway, with the ratio to total annotated DEGs in parentheses. The colored boxes highlight pathways of interest, including stress responses, light signaling, secondary metabolism, and carbon metabolism.

Based on the observation that *lmm64* mutant accumulates ROS and develops lesions under light conditions, we performed an intersection analysis of DEGs from three key comparisons, including *lmm64-L* vs WT-L, *lmm64-L* vs *lmm64-D*, and *lmm64-D* vs WT-D. A total of 907 DEGs were identified as being specifically associated with light-dependent lesion (**Figure 3A**). Among them, 750 genes displayed consistent expression trends (528 up-regulated and 222 down-regulated), while 157 genes with discordant patterns between comparisons were excluded (**Figure 3B**; **Supplementary Table 4**).

KEGG enrichment analysis revealed that the 750 lesion-associated DEGs were significantly enriched in pathways related to secondary metabolism, stress responses, light signaling and carbon metabolism (**Figure 3C**). Secondary metabolism was a prominently enriched category, mainly associated with phenylpropanoid biosynthesis (including flavonoid biosynthesis) and ubiquinone/terpenoid-quinone biosynthesis. Stress-related pathways included plant hormone signal transduction, protein processing in endoplasmic reticulum, MAPK signaling, and cysteine and methionine metabolism. Light signaling was primarily represented by circadian rhythm-plant. Carbon metabolism pathways encompassed glycolysis/gluconeogenesis, pyruvate metabolism, starch and sucrose metabolism, and galactose metabolism.

#### Phenylpropanoid/flavonoid biosynthetic genes

**Figure 4:**
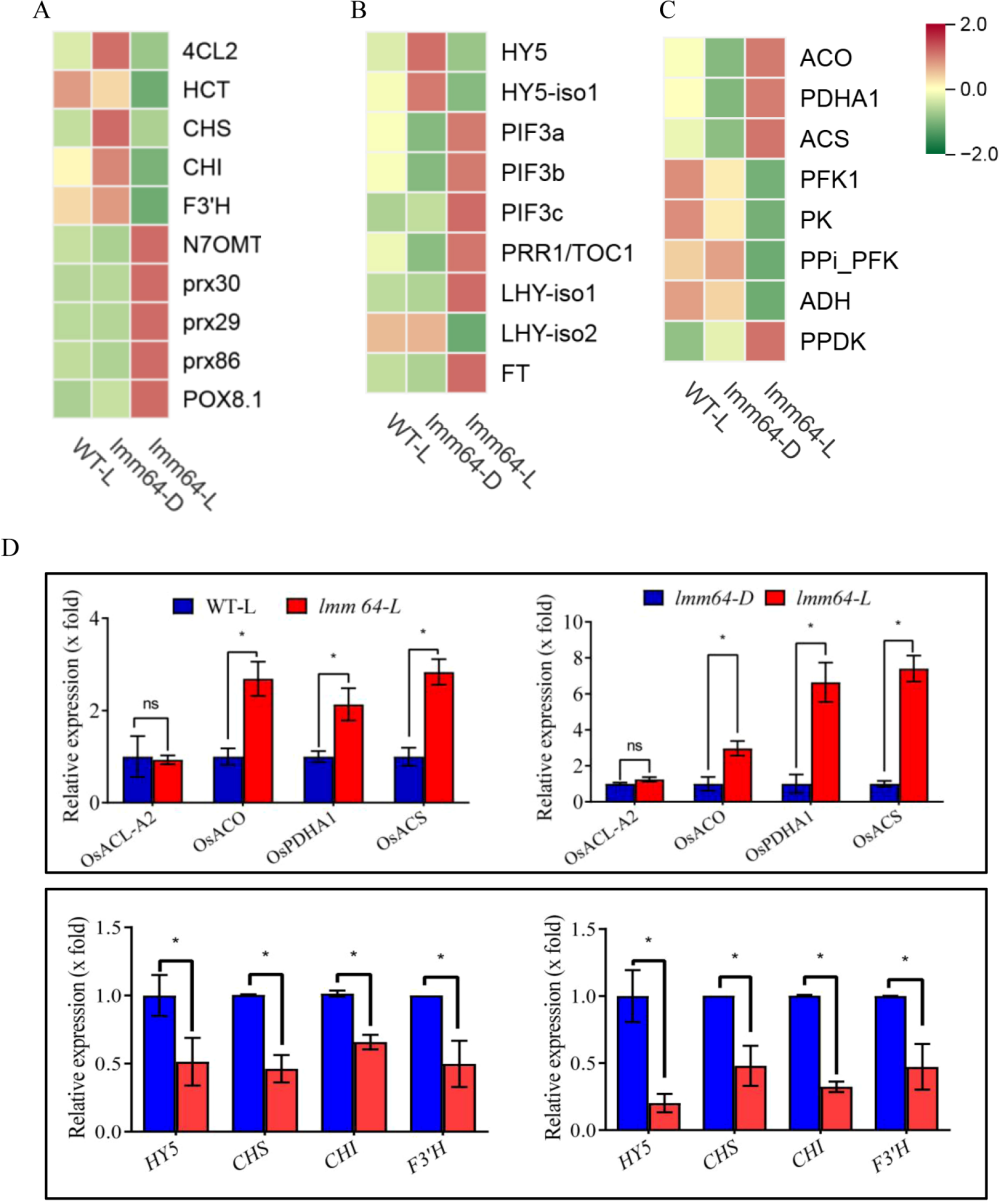
Transcriptional changes of phenylpropanoid/flavonoid biosynthesis, light signaling, and acetyl-CoA metabolism in *lmm64-L*. **(A–C)** Heatmaps showing expression of **(A)** phenylpropanoid/flavonoid biosynthetic genes, **(B)** core light signaling genes, and **(C)** acetyl-CoA metabolism-related genes in WT-L, *lmm64-D*, and *lmm64-L*. Values are Z-score normalized FPKM. Phenylpropanoid/flavonoid biosynthetic genes, including entry genes *4CL2* (*4-coumarate-CoA ligase 2*), *HCT* (*shikimate O-hydroxycinnamoyltransferase*), and core flavonoid genes *CHS* (*chalcone synthase*), *CHI* (*chalcone isomerase*), *F3’H* (*flavonoid 3’-hydroxylase*); *PRXs* (*peroxidases*); light signaling components *HY5* (*elongated hypocotyl 5*), *PIF3* (phytochrome-interacting factor 3), *LHY* (*late elongated hypocotyl*), *PRR1/TOC1* (*pseudo-response regulator 1/timing of cab expression 1*), and *FT* (*flowering locus T*); acetyl-CoA metabolism genes *ACO* (*aconitase*), *ACS* (*acetyl-CoA synthetase*), and *PDHA1* (*pyruvate dehydrogenase E1 alpha subunit*); glycolytic genes *PFK1* (*phosphofructokinase 1*), *PK* (*pyruvate kinase*), and *ADH* (*alcohol dehydrogenase*). **(D)** qRT-PCR validation of selected genes. Expression trends were consistent with RNA-seq data. Values are mean ± SD (n=3). *P < 0.05.

Phenylpropanoid/flavonoid biosynthesis was the top pathway within secondary metabolism (**Figure 3C**). Multiple genes across different steps showed altered expression within this pathway (**Figure 4A**; **Supplementary Table 5**). *4CL2* (*4-coumarate-CoA ligase*) and *HCT* (*shikimate O-hydroxycinnamoyltransferase*), key entry genes directing flux into the phenylpropanoid pathway, was downregulated. In the downstream flavonoid branch, core biosynthetic genes including *CHS* (*chalcone synthase*), *CHI* (*chalcone isomerase*), and *F3’H* (*flavonoid 3’-hydroxylase*) were also significantly downregulated in *lmm64-L* compared to controls. In contrast, four *PRXs* (*peroxidases*), which are not part of the flavonoid biosynthesis branch but function in downstream oxidative reactions within the phenylpropanoid pathway, were upregulated in *lmm64-L* (**Figure 4A**; **Supplementary Table 5**).

#### Stress-responsive genes

Multiple pathways involved in stress responses were abundantly enriched (**Supplementary Table 6**). The majority of stress-responsive genes within these pathways were upregulated in *lmm64-L*, including components of MAPK signaling (*MAPKKK*), defense regulators (*WRKY22*), ROS metabolism (*PRX*, *peroxidase*; *GST*, *glutathione S-transferase*), hormone signaling (*PYL8, pyrabactin resistance 1-like 8;, ERF1*, *ethylene response factor 1*), and stress perception (*RLK*, *receptor-like kinase*; *CML*, c*almodulin-like protein*; *HSP*, *heat shock protein*). Only a few genes, such as *MYC2* (*transcription factor MYC2*) and *EIN3* (ethylene-insensitive 3), showed downregulation. These results suggest that multiple stress-related pathways are co-activated and may be associated with cell death and ROS accumulation during lesion formation.

#### Light signaling genes

Two *HY5* genes (*elongated hypocotyl 5*), which positively regulate photomorphogenesis, were downregulated. Three *PIF3* (*phytochrome-interacting factor 3*) homologs, which act as negative regulators of photomorphogenesis, were upregulated. The circadian clock components *LHY* (*late elongated hypocotyl*) and *PRR1/TOC1* (*pseudo-response regulator 1/timing of cab expression 1*), as well as *FT* (*flowering locus t*), also showed altered expression, with a *LHY-isoform2* downregulated and the others upregulated (**Figure 4B**; **Supplementary Table 7**). The enrichment of light signaling genes among lesion-associated DEGs links them to the light-dependent phenotype of *lmm64*.

#### Acetyl-CoA metabolism-related genes

Because acetyl-CoA is the direct enzymatic product of OsACL, we examined genes involved in its metabolism within the carbon metabolism pathways, including those in glycolysis and the TCA cycle. *ACS* (*acetyl-CoA synthetase*) and *PDHA1* (*pyruvate dehydrogenase E1α*), involved in acetyl-CoA synthesis, and *ACO* (*aconitate hydratase*), which catalyzes citrate conversion in the TCA cycle, were all upregulated in *lmm64-L*. In contrast, glycolytic genes including *PFK1* (*6-phosphofructokinase*), *PK* (*pyruvate kinase*), and *ADH* (*alcohol dehydrogenase*) were downregulated (**Figure 4C**; **Supplementary Table 8**). These coordinated changes may be a metabolic adaptation to acetyl-CoA deficiency caused by OsACL dysfunction in the mutant.

qRT-PCR analysis confirmed the expression trends observed in RNA-seq for selected genes, including *CHS*, *CHI*, *F3’H*, *HY5*, *PIF3*, *ACO*, *ACS* and *PDHA1* (**Figure 4D**). Notably, *OsACL-A2* transcript levels showed no significant differences in *lmm64-L* compared to WT-L or *lmm64-D*, indicating that the mutation affects gene function at the post-transcriptional level.

### Metabolomic analysis reveals metabolic divergence in *lmm64* under light conditions

**Figure 5:**
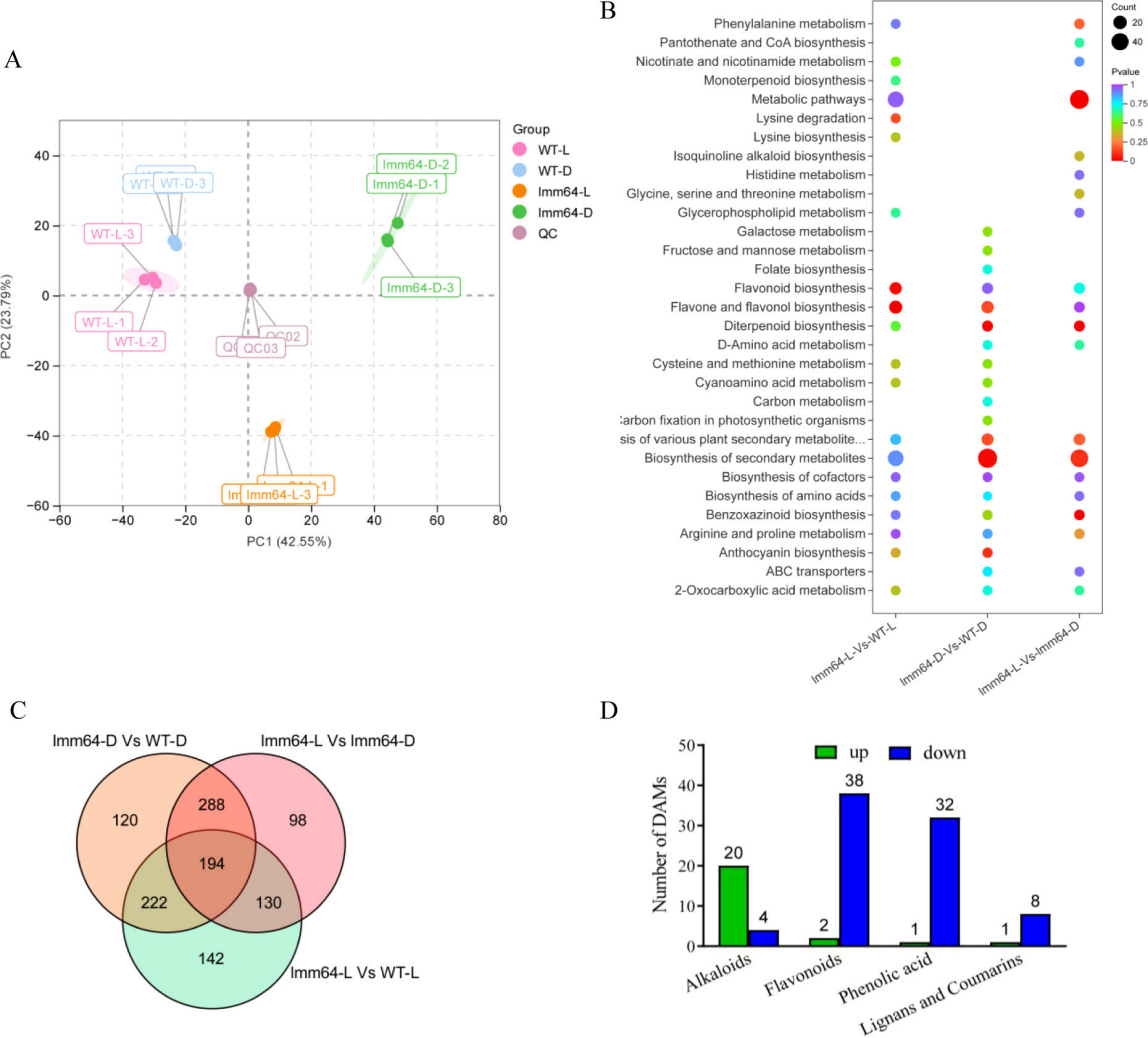
Metabolome profiling reveals metabolic divergence in *lmm64* under light conditions. **(A)** PCA score plot of metabolite profiles from each sample group and quality controls (QC). QC samples (pooled mixtures of all biological samples) cluster tightly together, indicating good instrument stability and data reproducibility throughout the analytical run. PC1 and PC2 indicate the first and second principal components, respectively, with percentages showing the explained variance. Each point represents an individual sample, colored by group. **(B)** KEGG enrichment analysis of DAMs identified from the comparisons of *lmm64-L* vs WT-L, *lmm64-D* vs WT-D, *lmm64-L* vs *lmm64-D*, and *lmm64-D* vs WT-D. Phenylpropanoid biosynthesis, glycerophospholipid metabolism, and nicotinate and nicotinamide metabolism were enriched. **(C)** Venn diagram showing identification of 130 lesion-associated metabolites based on significant changes in *lmm64-L* vs both WT-L and *lmm64-D*. **(D)** Classification of lesion-associated metabolites. Phenolic acids, lignans/coumarins, and flavonoids were predominantly downregulated, while alkaloids were upregulated in *lmm64-L*. Numbers indicate metabolite counts.

Given that transcriptome analysis revealed prominent enrichment of metabolic pathways among the 750 lesion-associated DEGs, we performed UPLC-MS/MS-based metabolomic analysis of *lmm64* and wild-type (WT) plants under light and dark conditions. Principal component analysis (PCA) showed that the first two principal components accounted for 66.34% of the total variance, clearly separating samples by genotype and light condition (**Figure 5A**). WT samples clustered tightly together regardless of light condition, whereas *lmm64* samples exhibited distinct clustering between light and dark treatments (**Supplementary Figure 3**), indicating that the *lmm64* mutation leads to extensive metabolic changes specifically under light.

To identify metabolic pathways associated with the light-dependent lesion phenotype, we performed KEGG enrichment analysis of DAMs from three key comparisons. The two comparisons involving lesion samples (*lmm64-L* vs WT-L and *lmm64-L* vs *lmm64-D*) showed similar enrichment patterns, including phenylpropanoid biosynthesis, glycerophospholipid metabolism, and nicotinate and nicotinamide metabolism (**Figure 5B**).

We further identified metabolites specifically associated with the lesion phenotype by Venn diagram analysis of 1,019 DAMs. A total of 130 metabolites were identified as lesion-associated, with significant changes in *lmm64-L* compared to both WT-L and *lmm64-D* (**Figure 5C**). Among these, 125 exhibited consistent trends across comparisons (27 up-regulated, 98 down-regulated), while the remaining 5 exhibited discordant patterns. Notably, phenolic compounds were predominantly downregulated, including 32/33 phenolic acids, 8/9 lignans and coumarins, and 38/40 flavonoids. In contrast, alkaloids were predominantly upregulated, with 20/24 identified alkaloids accumulating to higher levels in *lmm64-L* (**Figure 5D**; **Supplementary Figure 4**; **Supplementary Table 9**).

### Integrated analysis identifies phenylpropanoid/flavonoid pathway as a co-hub linking metabolic dysfunction to lesion formation

**Figure 6:**
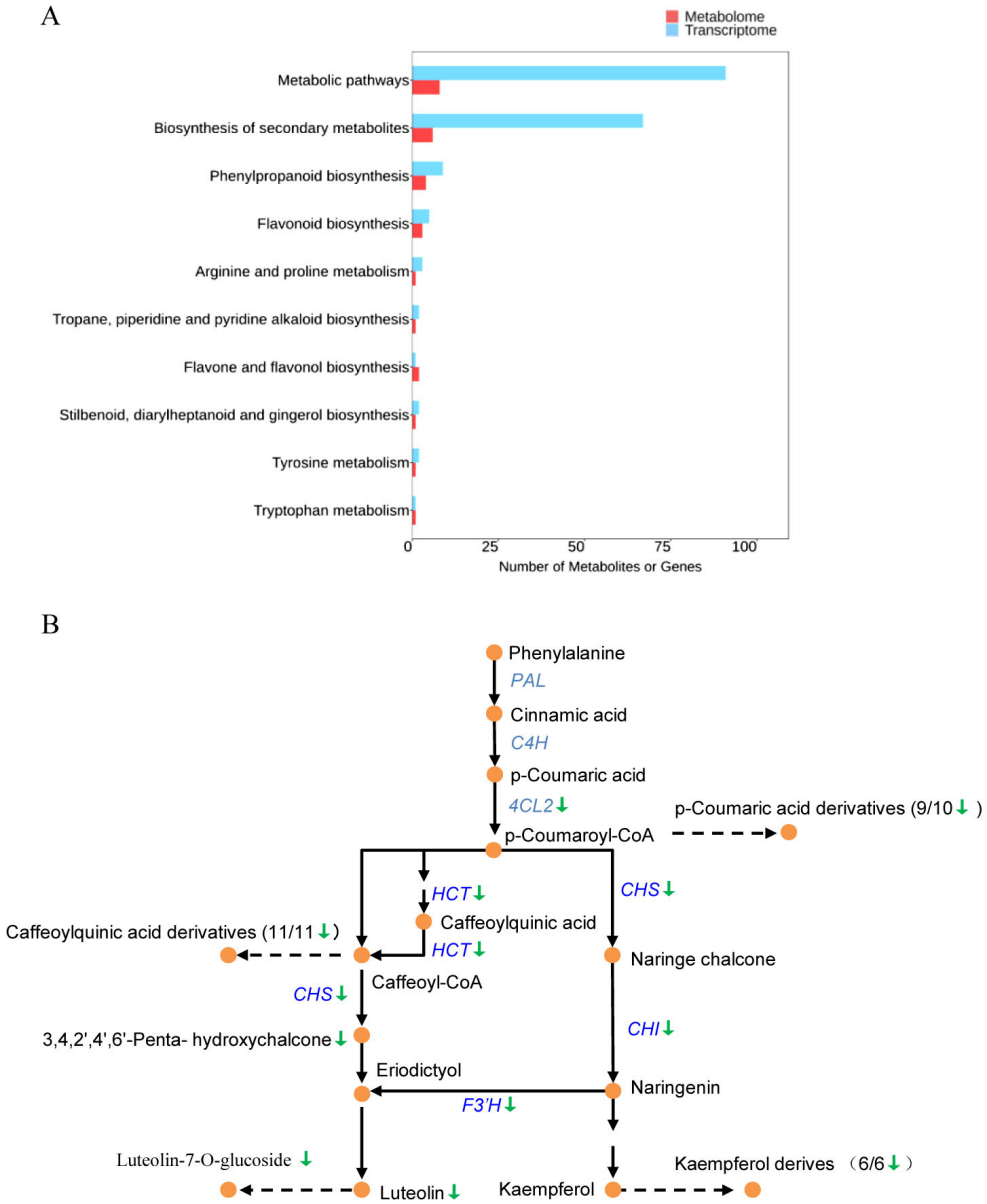
Integrated analysis of transcriptomic and metabolomic changes in the phenylpropanoid/flavonoid pathway. **(A)** KEGG co-enrichment analysis identifying phenylpropanoid/flavonoid biosynthesis as the most significantly shared pathway between 750 DEGs and 125 DAMs. **(B)** Schematic diagram of the phenylpropanoid/flavonoid pathway showing coordinated downregulation of genes and metabolites in *lmm64-L*. *Chalcone synthase, CHS*; *Chalcone isomerase, CHI*; *Flavonoid 3’-hydroxylase*, *F3’H*; *4-coumarate-CoA ligase*, *4CL*; *shikimate O-hydroxycinnamoyltransferase*, *HCT*. Green arrows indicate downregulation of genes (blue) or metabolites. Numbers in parentheses indicate downregulated/detected derivatives for each metabolite class.

To explore the relationship between transcriptional and metabolic changes in *lmm64-L*, we integrated the 750 DEGs with the 125 DAMs. KEGG co-enrichment analysis identified phenylpropanoid/flavonoid biosynthesis as the most significantly shared pathway (**Figure 6A**). Within this pathway, multiple biosynthetic genes were downregulated, including early genes *CHS*, *CHI*, and *F3’H*, as well as entry genes *4CL2* and *HCT*. Consistently, their corresponding metabolites showed reduced accumulation. These included the direct CHS product 3,4,2’,4’,6’-Pentahydroxychalcone, downstream flavonoids such as luteolin glycosides (e.g., Luteolin-7-O-glucoside) and kaempferol derivatives (e.g., Kaempferol-4’-O-glucoside, Tiliroside), and phenolic acid derivatives including caffeoylquinic acid isomers (Chlorogenic acid, Cryptochlorogenic acid) and p-coumaric acid conjugates (p-Coumaroyl Aminogalactitol, 1-O-p-Coumaroyl-β-D-glucose) (**Figure 6B**; **Supplementary Table 9**). These coordinated changes indicate systemic suppression of phenylpropanoid/flavonoid biosynthesis in *lmm64-L*.

## Discussion

Lesion mimic phenotypes, characterized by spontaneous cell death, are caused by mutations in numerous genes through diverse molecular mechanisms across plant species, highlighting the complexity of programmed cell death regulation. In rice, *OsACL-A2* has been consistently identified as a causal gene for lesion formation in both previous studies and our study (Ruan et al., 2019; Duan et al., 2025; **Figure 1C**). Ruan et al. (2019) revealed that *OsACL-A2* mutation leads to degradation of its protein through the ubiquitin-proteasome pathway, accompanied by activation of defense-related genes. Duan et al. (2025) showed that *OsACL-A2* mutation disrupts iron homeostasis, further expanding our understanding of how this gene contributes to lesion development. In this study, integrated transcriptomic and metabolomic analyses were utilized to further explore the specific and common transcriptional and metabolic changes associated with lesion mimic phenotypes.

Lesion mimic phenotypes are caused by programmed cell death, a process characterized by ROS accumulation, chloroplast disruption, and activation of defense- and cell death-related genes (Chen et al., 2025; Tian et al., 2025). Indeed, transcriptomic analysis of the lesion-forming *lmm64* mutant under light revealed significant enrichment of defense- and cell death-related genes and chloroplast components such as the photosynthetic membrane (**Figure 2A**). Notably, defense- and cell death-related genes were extensively enriched regardless of light condition (**Figure 2B**), indicating a pre-activated defense state in the mutant even in darkness, a phenomenon also reported in the *les30* mutant (Li et al., 2024). Furthermore, we focused on 750 genes specifically associated with the lesion phenotype (**Figure 3B**; **Supplementary Table 4**). Among these, several defense- or cell death-related genes exhibited altered expression, including components of MAPK signaling (*MAPKKK*), defense regulators (*WRKY22*), ROS metabolism (*PRX*, *GST*), hormone signaling (*PYL8*, *ERF1*, *MYC2*, *EIN3*), and stress perception (*RLK*, *CML*, *HSP*) (**Supplementary Table 6**). These results indicate that defense-related gene regulation in *lmm64* operates at two levels: a basal activation independent of light, and a light-dependent subset whose expression changes are closely associated with lesion formation.

ROS accumulation often results from impaired ROS-scavenging capacity, reflected by alterations in antioxidant enzyme activities, including catalase (CAT), superoxide dismutase (SOD), and peroxidase (PRX). However, the patterns of these alterations are complex and context-dependent in lesion mimic mutants (Al Amin et al., 2019; Zheng et al., 2021; Xia et al., 2024). Reduced CAT activity has been reported in the *OsACL-A2* mutant, indicating that disruption of this gene leads to impaired ROS scavenging capacity. In our study, transcriptomic analysis revealed that almost *CAT* and *SOD* genes showed no significant changes in *lmm64-L* compared to WT-L, and their expression alterations in other comparisons lacked lesion-specific association (**Supplementary Table 11**). The discrepancy between our transcriptomic data (unchanged CAT/SOD expression) and the reduced CAT activity reported previously may reflect post-transcriptional or translational regulation. In contrast, four *PRXs* were consistently upregulated under lesion-inducing conditions (**Figure 4A**; **Supplementary Table 5**). Whether the transcriptional upregulation of these PRX genes translates to corresponding changes at the enzymatic level requires further investigation.

KEGG pathway analysis revealed significant enrichment of secondary metabolite biosynthesis genes among the lesion-associated DEGs, particularly those involved in phenylpropanoid and flavonoid pathways (**Figure 3C**). These metabolites function as crucial antioxidants (Treutter, 2006; Ramaroson et al., 2022). Previous transcriptomic study showed that the large families of phenylpropanoid biosynthetic genes like *PAL*, *HCT*, and *caffeic acid O-Methyltransferase* (*COMT*) and the flavonoid biosynthetic genes like *CHS*, *DFR*, *F3’H*, and *FLS* were upregulated in lesion mimic mutants (Mu et al., 2021; Li et al., 2022), suggesting their potential role in modulating ROS levels during lesion formation. In contrast, *lmm64-L* exhibited a distinct pattern. Transcriptomic analysis revealed consistent downregulation of phenylpropanoid/flavonoid core biosynthetic genes, including *4CL*, *HCT*, *CHS*, *CHI*, and *F3’H* (**Figure 4A**; **Supplementary Table 5**), which was corroborated by metabolomic data showing significant depletion of antioxidant flavonoids and phenolic acids (**Figure 5D**; **Figure 6B**; **Supplementary Figure 4**; **Supplementary Table 9**). These results indicate a distinct regulatory mechanism underlying flavonoid metabolism in *lmm64*. Whether the reduction in phenylpropanoid/flavonoid metabolites affects ROS scavenging capacity remains to be verified.

While previous studies have reported enrichment of photosynthesis-related genes in lesion mimic mutants (Mu et al., 2021; Li et al., 2022), our analysis revealed prominent enrichment of core light signaling components (*HY5*, *PIF3*, *LHY*, *PRR1/TOC1* and *FT*), alongside a small subset of photosynthesis-related genes including *ferredoxin*, *photosystem II 10kDa protein* (*PsbR*), and c*oproporphyrinogen III oxidase* (*CPOX*), among the lesion-associated genes identified in *lmm64-L* (**Figure 4B**; **Supplementary Table 7**).This suggests that reprogramming of light signaling genes, rather than photosynthesis-related changes alone, may play a key role in light-induced lesion formation.

Given downregulation of genes in the phenylpropanoid/flavonoid pathway in *lmm64-L*, we further investigated potential upstream regulators that might contribute to this reduction. Our analysis pointed to two upstream factors that likely contribute to downregulation of flavonoid pathways. First, light signaling components were markedly altered, particularly downregulation of *HY5* and upregulation of *PIFs* (**Figure 4B**; **Supplementary Table 7**). HY5, whose function is antagonized by PIFs, is a known transcriptional activator of flavonoid pathway genes (Xiao et al., 2022; Lin et al., 2025). Meanwhile, these two transcriptional regulators are differentially modulated by light perception through distinct signaling modes. PIFs directly interact with phytochromes (phyA, phyB) in a light-dependent manner, leading to their degradation and release of photomorphogenic repression (Castillon et al., 2009). In contrast, HY5 is indirectly regulated through the COP1-SPA complex downstream of multiple photoreceptors including phyB, cryptochromes (crys), and UV RESISTANCE LOCUS 8 (UVR8) (Toledo-Ortiz et al., 2014). Future studies with different light qualities and photoreceptor mutants would help elucidate the specific pathways mediating this response.

Second, the upregulation of acetyl-CoA-related genes, including *ACO*, *ACS*, and *PDHA1* (**Figure 4C**; **Supplementary Table 8**), likely represents a compensatory response to acetyl-CoA deficiency resulting from ACL dysfunction. Such a deficiency could in turn limit the production of malonyl-CoA, an essential acetyl-CoA-derived precursor that serves as the extender unit for flavonoid skeleton synthesis (Zhou et al., 2024). Unlike previously reported lesion mimic mutants where phenylpropanoid/flavonoid pathways are upregulated in many cases (Mu et al., 2021), the convergence of impaired light signaling and compromised precursor supply in *lmm64* likely underlies the observed reduction in these compounds.

**Figure 7:**
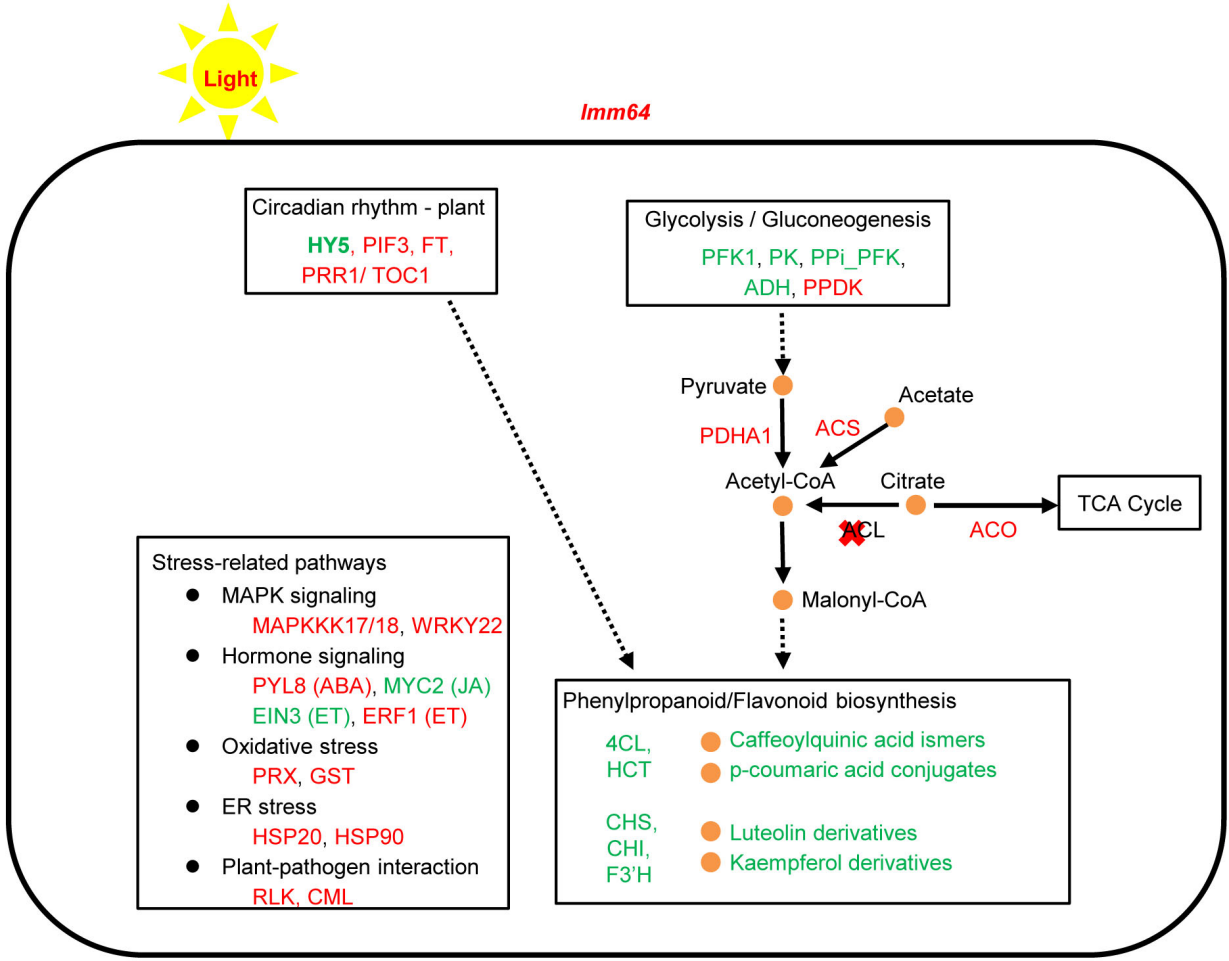
A working model for lesion formation under light in *lmm64* mutant. *OsACL-A2* mutation triggers concurrent alterations in acetyl-CoA metabolism, light signaling, and stress pathways, accompanied by downregulation of phenylpropanoid/flavonoid biosynthesis and depletion of antioxidant phenolic compounds. Orange dots: metabolites; red/green text: up/downregulation based on transcriptomic and metabolomic data; dashed arrows: multi-step processes. *Chalcone synthase, CHS*; *Chalcone isomerase*, *CHI*; *Flavanone 3’-hydroxylase*, *F3’H*; 4-coumarate-CoA ligase, *4CL*; *shikimate O-hydroxycinnamoyltransferase*, *HCT*. *Mitogen-Activated Protein Kinase Kinase Kinase*, *MAPKKK*; *Pyrabactin Resistance-like 8*, *PYL8*; *Heat Shock Protein, HSP*; *WRKY Transcription Factor 22*, *WRKY22*; *MYC2 Transcription Factor*, *MYC2*; *Peroxidase*, *PRX*; *Glutathione S-Transferase*, *GST*; *Ethylene Insensitive 3*, *EIN3*; *Ethylene Response Factor 1, ERF1*; *Receptor-Like Kinase, RLK*; *Calmodulin-Like Protein*, *CML*; Abscisic Acid, ABA; ET, Ethylene; Jasmonic Acid, JA.

In summary, *OsACL-A2* mutation in *lmm64* triggers concurrent alterations in acetyl-CoA metabolism, light signaling components, and stress-responsive pathways, accompanied by downregulation of the phenylpropanoid/flavonoid pathway and depletion of antioxidant flavonoids and phenolic acids (**Figure 7**). These molecular events occur specifically under light conditions and are closely associated with lesion formation, extending the functional landscape of *OsACL-A2* in rice.

## Data Availability

The transcriptome raw data have been deposited in NCBI BioProject under accession number PRJNA1444994. The processed metabolomics data supporting the conclusions of this study are available from the corresponding author upon reasonable request.

## Supplementary material

Supplementary Figure 1 Phenotypic and genetic characterization of *lmm64*. **(A)** Trypan blue staining of leaves from WT and *lmm64*. **(B)** DAB staining of leaves from WT and *lmm64*. **(C)** Comparisons between WT and *lmm64* in 1000-grain weight, number of effective panicles per plant, seed-setting rate, and number of seeds per panicle. Error bars represent SD (n = 10). Asterisks indicate significant differences at P < 0.05 by Student’s t-test. **(D)** Fine mapping of *lmm64* locus. The gene was delimited to an approximately 30 kb genomic region between markers B18.95 and B18.126 on chromosome 12.

Supplementary Figure 2 Transcriptome data quality and DEG analysis. (A) Inter-sample correlation heatmap displaying pairwise Pearson correlation coefficients (r). The color gradient from light yellow (r ~ 0) to deep red (r ~ 1) indicates the strength of the positive correlation. (B) Bar plot showing the number of DEGs identified in different comparative groups. Upregulated and downregulated genes are represented by blue and green bars, respectively.

Supplementary Figure 3 Hierarchical clustering of metabolite profiles. Hierarchical clustering heatmap of metabolite abundances. Columns represent samples; rows represent metabolites clustered by expression patterns. Color intensity reflects relative abundance levels.

Supplementary Figure 4 Lesion-associated DAMs in *lmm64-L*. Hierarchical clustering heatmap of DAM abundances identified from comparisons involving lesioned (*lmm64-L*) and non-lesioned samples. DAMs are categorized into several classes including alkaloids, flavonoids, and phenolic acids. Columns represent individual samples; rows represent metabolites clustered by expression patterns. Color intensity reflects relative abundance levels.

Supplementary Table 1 Primers used in this study.

Supplementary Table 2 Genomic variation analysis of the *OsACL-A2* locus and flanking 2-kb regions in diverse rice varieties.

Supplementary Table 3 Summary of sequencing quality metrics.

Supplementary Table 4 List of 907 lesion-associated DEGs identified in *lmm64-L*.

Supplementary Table 5 Lesion-associated DEGs in secondary metabolism pathways.

Supplementary Table 6 Lesion-associated DEGs in stress-related pathways pathways.

Supplementary Table 7 Lesion-associated DEGs in light signaling- and photosynthesis-related pathways.

Supplementary Table 8 Lesion-associated DEGs in carbon metabolism pathways.

Supplementary Table 9 List of 130 DAMs associated with lesion formation in *lmm64-L*.

Supplementary Table 10 Lesion-associated DAMs associated with phenylpropanoid/flavonoid metabolites and derivatives in *lmm64-L*.

Supplementary Table 11 Several well-characterized ROS-related genes.
