# Supplementary figures and images for: Transcriptomic and metabolomic analyses reveal OsACL-A2 coordinates light signaling, stress responses, and metabolic changes in rice
Source: Front Plant Sci. 2026 Apr 10;17:1789851. doi: 10.3389/fpls.2026.1789851 (PMC13105920; doi:10.3389/fpls.2026.1789851)

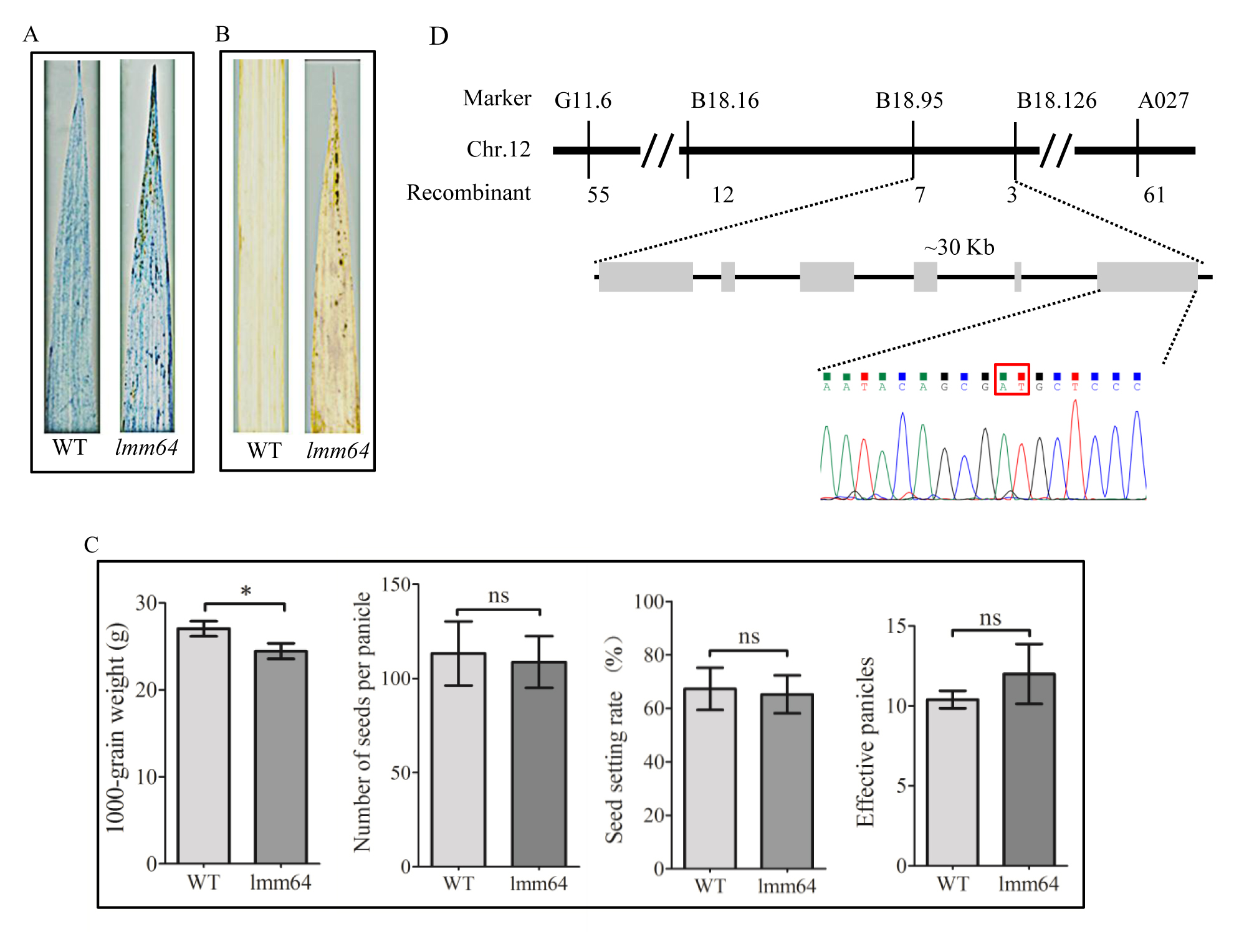

Supplement: Supplementary Figure 1 — Phenotypic and genetic characterization of lmm64. (A) Trypan blue staining of leaves from WT and lmm64. (B) DAB staining of leaves from WT and lmm64. (C) Comparisons between WT and lmm64 in 1000-grain weight, number of effective panicles per plant, seed-setting rate, and number of seeds per panicle. Error bars represent SD (n = 10). Asterisks indicate significant differences at P < 0.05 by Student’s t-test. (D) Fine mapping of lmm64 locus. The gene was delimited to an approximately 30 kb genomic region between markers B18.95 and B18.126 on chromosome 12. [file Image1.jpeg]

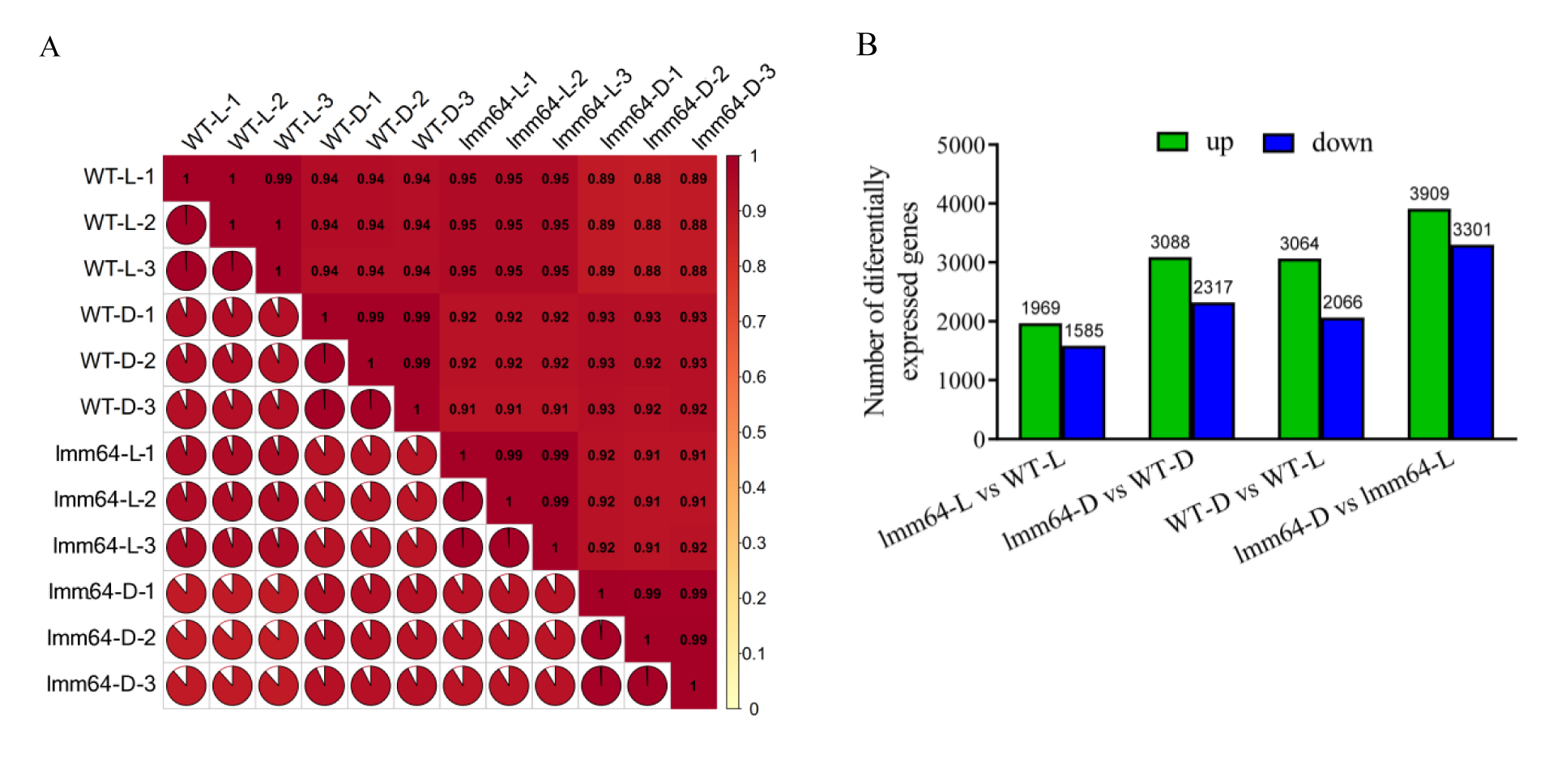

Supplement: Supplementary Figure 2 — Transcriptome data quality and DEG analysis. (A) Inter-sample correlation heatmap displaying pairwise Pearson correlation coefficients (r). The color gradient from light yellow (r ~ 0) to deep red (r ~ 1) indicates the strength of the positive correlation. (B) Bar plot showing the number of DEGs identified in different comparative groups. Upregulated and downregulated genes are represented by blue and green bars, respectively. [file Image2.jpeg]

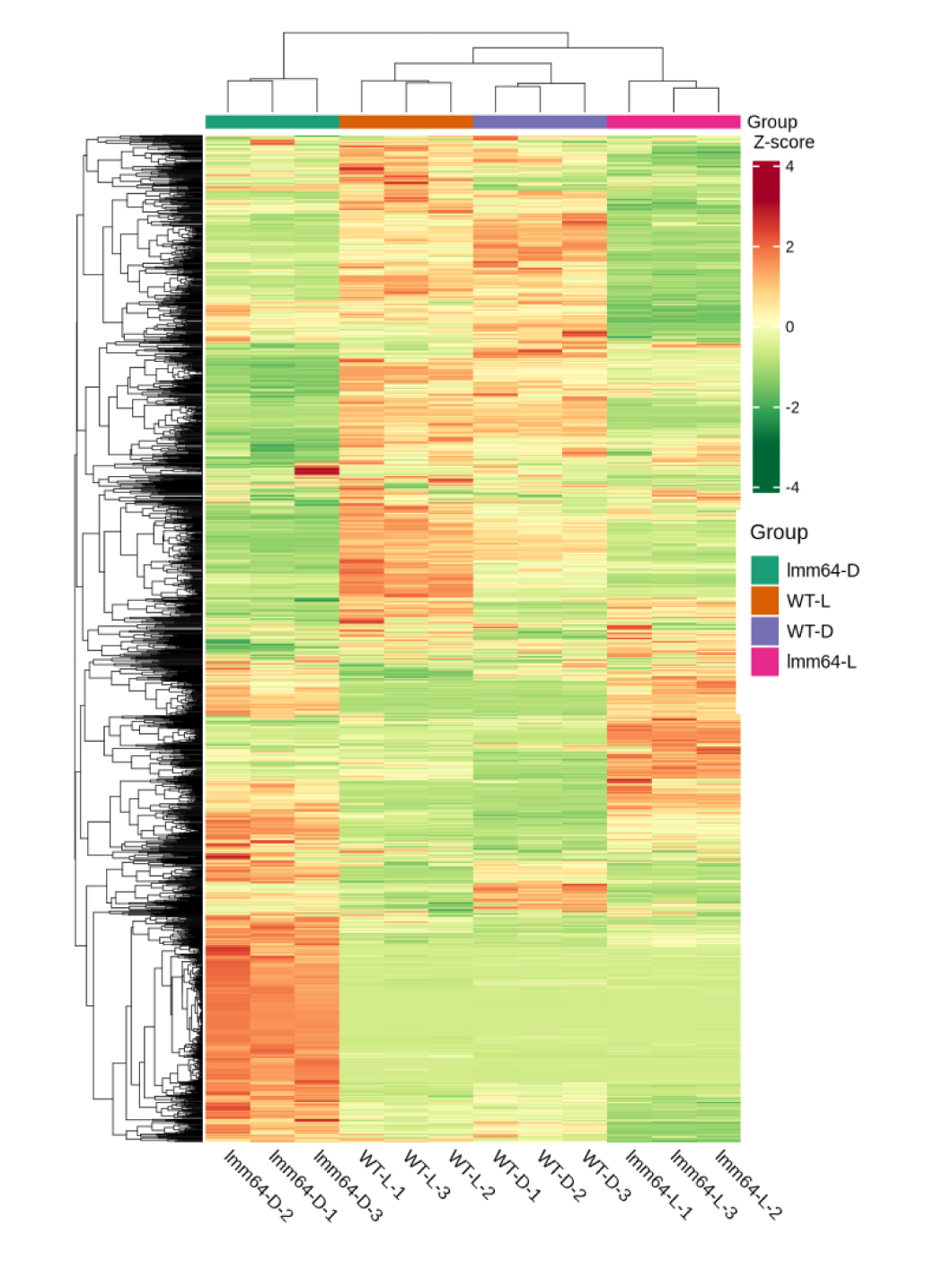

Supplement: Supplementary Figure 3 — Hierarchical clustering of metabolite profiles. Hierarchical clustering heatmap of metabolite abundances. Columns represent samples; rows represent metabolites clustered by expression patterns. Color intensity reflects relative abundance levels. [file Image3.jpeg]

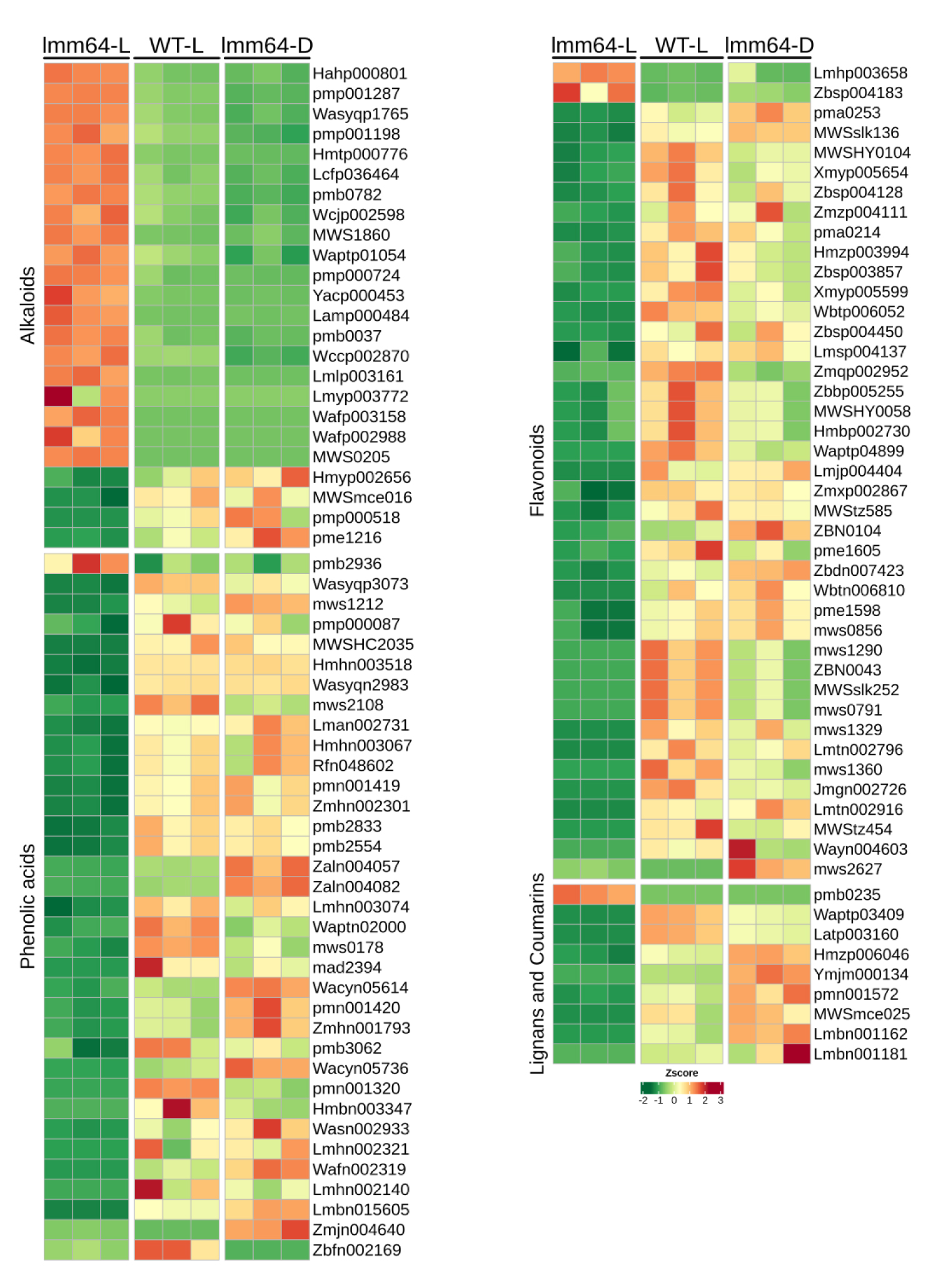

Supplement: Supplementary Figure 4 — Lesion-associated DAMs in lmm64-L. Hierarchical clustering heatmap of DAM abundances identified from comparisons involving lesioned (lmm64-L) and non-lesioned samples. DAMs are categorized into several classes including alkaloids, flavonoids, and phenolic acids. Columns represent individual samples; rows represent metabolites clustered by expression patterns. Color intensity reflects relative abundance levels. [file Image4.jpeg]
